# Supplementary material for: Predictors of Post-Traumatic Stress Symptoms after musculoskeletal trauma
Source: PLoS One. 2026 May 6;21(5):e0348595. doi: 10.1371/journal.pone.0348595 (PMC13148695; doi:10.1371/journal.pone.0348595)
Supplement: S1 File — (DOCX) [file pone.0348595.s001.docx]

**Supplementary file 1**

**File 1: STROBE reporting guideline**

STROBE Statement—checklist of items that should be included in reports of observational studies.

|  | **Item No.** | **Recommendation** | **Page  No.** | **Relevant text from manuscript** |
| --- | --- | --- | --- | --- |
| **Title and abstract** | 1 | (*a*) Indicate the study’s design with a commonly used term in the title or the abstract | Title and page 1 | This was a retrospective cohort study using data from a prospective observational study of patients aged 16 and older admitted to a trauma centre in the UK for acute musculoskeletal trauma. |
|  |  | (*b*) Provide in the abstract an informative and balanced summary of what was done and what was found | 2-3 | 1. This was a retrospective cohort study using data from a prospective observational study of patients aged 16 and older admitted to a trauma centre in the UK for acute musculoskeletal trauma. 2. At the 3-month follow-up, falls and road traffic accidents were associated with an increased risk of developing PTSS, while higher anxiety and fear of movement scores were linked to a reduced risk. 3. At 6 months, higher disability scores were associated with a lower risk of PTSS. 4. Injury severity significantly impacts PTSS severity, with differences between ‘Mild’ and ‘Moderate’ or ‘Major' levels. 5. This information is essential for tailoring rehabilitation strategies and clinical care for musculoskeletal trauma patients." |
| **Introduction** | | | |  |
| Background/rationale | 2 | Explain the scientific background and rationale for the investigation being reported | 4-5 | 1. In recent years, there has been growing interest in studying the psychological effects of musculoskeletal injuries and the development of PTSS. 2. The literature review offers valuable insights into the complex relationship between musculoskeletal injuries and PTSS. Despite these limitations, the studies consistently emphasise the significant impact of injury severity, pain, disability, and psychological factors on the development and persistence of PTSS. |
| Objectives | 3 | State specific objectives, including any prespecified hypotheses | 5 | This study aims to determine predictors of PTSS at three and six months post-musculoskeletal trauma and hospitalisation by examining socio-demographic factors, pre-existing mental health conditions, social support, coping strategies, and injury severity. |
| **Methods** | | | |  |
| Study design | 4 | Present key elements of study design early in the paper | Page 1 in the abstract | This was a retrospective cohort study using data from a prospective observational study of patients aged 16 and older admitted to a trauma centre in the UK for acute musculoskeletal trauma. |
| Setting | 5 | Describe the setting, locations, and relevant dates, including periods of recruitment, exposure, follow-up, and data collection | 6 | A description of the data collection is provided in detail elsewhere [19]. In brief, data were analysed from consecutive patients with acute musculoskeletal trauma aged 16 years and older who were emergency admissions in a major trauma centre in the West Midlands, United Kingdom. The hospital admission register was used to identify all consecutive eligible patients between December 2018 and March 2020. |
| Participants | 6 | **(*a*) *Cohort study*—Give the eligibility criteria, and the sources and methods of selection of participants. Describe methods of follow-up**  *Case-control study*—Give the eligibility criteria, and the sources and methods of case ascertainment and control selection. Give the rationale for the choice of cases and controls  *Cross-sectional study*—Give the eligibility criteria, and the sources and methods of selection of participants | 6 | Full details of the original study can be found in Evans et al. [19], which included patients who were admitted to the trauma centre within the previous 14 days due to acute musculoskeletal trauma. Patients were also required to be able to understand and use written and spoken English, as well as have the mental capacity to provide informed consent (e.g., no confusion, delirium, severe cognitive impairment, or severe mental illness, defined by a score of ≤6 on the Abbreviated Mental Test [AMT-10]) [20]. Patients were excluded if they had acute intracranial bleeding and a Glasgow Coma Scale score of 14 or less, brain or central nervous system injury, long-term neurocognitive disorders (e.g., brain tumour, multiple sclerosis, Alzheimer’s disease, or Parkinson’s disease), ongoing rheumatological condition, prolonged corticosteroid use, comorbid cancer, or terminal illness with short life expectancy. |
|  |  | (*b*) *Cohort study*—For matched studies, give matching criteria and number of exposed and unexposed  *Case-control study*—For matched studies, give matching criteria and the number of controls per case | N/A  N/A | N/A  N/A |
| Variables | 7 | Clearly define all outcomes, exposures, predictors, potential confounders, and effect modifiers. Give diagnostic criteria, if applicable | 7 | Potential predictors measured at baseline, three months, and six months were analysed, as these time points provided sufficient information for meaningful interpretation. The 12-month data were excluded due to significant missingness, rendering multiple imputation unreliable for robust analysis. Clinical variables were measured consistently at three and six months. A wide range of variables were included to capture a comprehensive set of potential predictors. Potential predictors were identified based on previous studies [25, 26]. The complete list of candidate predictors is shown in Supplementary file 2. |
| Data sources/ measurement | 8* | For each variable of interest, give sources of data and details of methods of assessment (measurement). Describe comparability of assessment methods if there is more than one group | 6 | A description of the data collection is provided in detail elsewhere [19]. In brief, data were analysed from consecutive patients with acute musculoskeletal trauma aged 16 years and older who were emergency admissions in a major trauma centre in the West Midlands, United Kingdom. The hospital admission register was used to identify all consecutive eligible patients between December 2018 and March 2020. See above information |
| Bias | 9 | Describe any efforts to address potential sources of bias | 7-8 | This study uses multivariate analysis to reduce sources of bias, ensuring a more accurate identification of predictors associated with PTSS. |
| Study size | 10 | Explain how the study size was arrived at | 5-6 | The study included 125 participants and 36 variables. Ideally, there should be at least 10 times the number of events as the number of candidate predictors, which would mean 360 events are needed. This study faced challenges in reaching the recommended 10 events per candidate predictor due to high attrition rates and the nature of musculoskeletal injuries, which hindered participants’ full engagement with the study. As a result, the study utilised the available data while acknowledging its limitations. |

| Quantitative variables | 11 | Explain how quantitative variables were handled in the analyses. If applicable, describe which groupings were chosen and why |  | The strength of each potential PTSS risk predictor was tested separately using linear regression. A significance level of (p<0.05) was chosen to indicate a potential association between PTSS and the candidate predictor. Regression coefficient including their 95% confidence intervals (CIs) were reported. Any predictor variable that met the p<0.05 threshold was selected for inclusion in the multivariate analysis. Patients were grouped by the severity of their injuries: mild, moderate, and major. The Kruskal-Wallis’s test was used to compare changes in PTSS severity at 3- and 6-months follow-up after musculoskeletal trauma. |
| --- | --- | --- | --- | --- |
| Statistical methods | 12 | (*a*) Describe all statistical methods, including those used to control for confounding | 8-10 | Descriptive statistics were used to summarise participant characteristics, including means, standard deviations (SD), medians, and interquartile ranges (IQR) for continuous variables, and frequencies for categorical variables. Linear regression tested each PTSS risk predictor. Predictors with p<0.05 were included in the multivariate analysis. A stepwise backward selection process was used to retain only significant predictors (p<0.05). Patients were grouped by injury severity: mild, moderate, and major. The Kruskal-Wallis’s test compared PTSS severity changes at 3- and 6-months post-trauma. |
|  |  | (*b*) Describe any methods used to examine subgroups and interactions | 10 | Patients were grouped by injury severity: mild, moderate, and major. The Kruskal-Wallis’s test compared PTSS severity changes at 3- and 6-months post-trauma. |
|  |  | (*c*) Explain how missing data were addressed | 7 | Missing data for variables like BMI and PROMs were addressed using multiple imputation (m=5) with the Multivariate Imputation by Chained Equations (MICE) technique, replacing missing values with plausible estimates to retain statistical power and ensure realistic regression coefficients. |
|  |  | (*d*) *Cohort study*—If applicable, explain how loss to follow-up was addressed  *Case-control study*—If applicable, explain how matching of cases and controls was addressed  *Cross-sectional study*—If applicable, describe analytical methods taking account of sampling strategy | 7 | Same as above |
|  |  | (*e*) Describe any sensitivity analyses | N/A | N/A |
| **Results** | | | | |
| Participants | 13* | (a) Report numbers of individuals at each stage of study—e.g., numbers potentially eligible, examined for eligibility, confirmed eligible, included in the study, completing follow-up, and analysed | 10 | Description of participants characteristics |
|  |  | (b) Give reasons for non-participation at each stage | 10 | Description of participants characteristics |
|  |  | (c) Consider use of a flow diagram | N/A | N/A |
| Descriptive data | 14* | (a) Give characteristics of study participants (e.g., demographic, clinical, social) and information on exposures and potential confounders | 10 | A total of 125 participants with a mean (SD) age of 48.9 ±18.7 years were included in this study. Follow-up response rates were 73 participants (58.9%) at three months, 82 participants (66.1%) at 6 months, and 44 participants (35.5%) at 12 months. In the current study, participant data were utilised only for the three- and six-month timepoints. The significantly lower response rate at the 12-month mark (35.5%) raised concerns about data completeness and the potential for introducing bias and uncertainty associated with imputation in a context where the dropout rate is high. Table 1 presents the baseline characteristics of the 125 participants. |
|  |  | (b) Indicate number of participants with missing data for each variable of interest | 10-12 | Presented in table 1: participant demographics |
|  |  | (c) *Cohort study*—Summarise follow-up time (e.g., average and total amount) | 10-12 | Presented in table 1: participant demographics |
| Outcome data | 15* | *Cohort study*—Report numbers of outcome events or summary measures over time | N/A | Supplementary files |
|  |  | *Case-control study—*Report numbers in each exposure category, or summary measures of exposure | N/A | N/A |
|  |  | *Cross-sectional study—*Report numbers of outcome events or summary measures | N/A | N/A |
| Main results | 16 | (*a*) Give unadjusted estimates and, if applicable, confounder-adjusted estimates and their precision (eg, 95% confidence interval). Make clear which confounders were adjusted for and why they were included | 1 | At the three month follow-up, falls (OR: 2.20, 95% CI 1.68 to 3.15, p=0.015) and road traffic accidents (RTAs) (OR: 3.71, 95% CI 2.60 to 6.85, p=0.010) as the causes of injury were associated with increased odds of having PTSS, while higher anxiety (OR: 0.53, 95% CI 0.33 to 0.86, p=0.011) and fear of movement (OR: 0.58, 95% CI 0.39 to 0.86, p=0.007) were linked to lower odds of experiencing PTSS. At six months, higher disability scores were associated with lower odds of having PTSS (OR: 0.89, 95% CI 0.82 to 0.97, p=0.013). At six months, PTSS scores varied by injury severity, with significant differences between injuries categorised as ‘Mild’ and ‘Moderate’ (p=0.018) and ‘Mild’ and ‘Major’ (p=0.032) severity. |
|  |  | (*b*) Report category boundaries when continuous variables were categorized | N/A | Supplementary files |
|  |  | (*c*) If relevant, consider translating estimates of relative risk into absolute risk for a meaningful time period | N/A | N/A |
