# Supplementary material for: Predictors of Post-Traumatic Stress Symptoms after musculoskeletal trauma
Source: PLoS One. 2026 May 6;21(5):e0348595. doi: 10.1371/journal.pone.0348595 (PMC13148695; doi:10.1371/journal.pone.0348595)
Supplement: S2 File — (DOCX) [file pone.0348595.s002.docx]

**Supplementary file 2: list of candidate predictors**

| **Clinical** | **Trauma** | **Social Demographics** |
| --- | --- | --- |
| Body Mass Index | Trauma description | Civilian/Military |
| Lowest Glascow Coma Scale | Upper limb injury | Alcohol consumption |
| Surgery | Lower limb injury | Smoking status |
| SF-36 physical | Back neck injury | Drug use |
| SF-36 mental | Chest abdominal injury | Medical history |
| EQ-5D-5L | Head face injury | Comorbidities |
| TSK-11 | Number of fractures | Days in hospital |
| HADS depression score | Number of surgeries | Age |
| HADS anxiety score | Injury severity | Gender |
| BPI Pain intensity | Previous surgery | Days since admission |
| IES-R score |  | Days since trauma |
| PSEQ |  | Education status |
| CPGS |  | Work status |
|  |  | Ethnic group |

**Abbreviation:** SF: Short Form; TSK: Tampa Scale of Kinesiophobia; HADS: Hospital Anxiety and Depression Scale; IES-R: Impact OF Events Scale-Revised
