# Supplementary material for: Predictors of Post-Traumatic Stress Symptoms after musculoskeletal trauma
Source: PLoS One. 2026 May 6;21(5):e0348595. doi: 10.1371/journal.pone.0348595 (PMC13148695; doi:10.1371/journal.pone.0348595)
Supplement: S3 File — (DOCX) [file pone.0348595.s003.docx]

Supplementary file 3: **Predictor Variables and Their Descriptions**

| **Predictor Variable** | **Type** | **Description** | **Coding/Measurement** | **Source** |
| --- | --- | --- | --- | --- |
| **Clinical Variables** | | | | |
| Body Mass Index (BMI) | Continuous | A measure of body fat based on height and weight. | Calculated as weight (kg) / height (m)^2 | Clinical assessment |
| Lowest Glasgow Coma Scale (GCS) | Continuous | The lowest score recorded during hospitalisation indicating the patient's level of consciousness. | Score from 3 (deep coma) to 15 (fully alert) | Clinical assessment |
| Surgery | Binary | Indicates whether the patient underwent surgery (yes/no). | Yes (1), No (0) | Clinical records |
| SF-36 Physical Functioning | Continuous | A score from the SF-36 questionnaire assessing physical health. | Scale from 0 (poor) to 100 (excellent) | Validated questionnaire |
| SF-36 Mental Health | Continuous | A score from the SF-36 questionnaire assessing mental health. | Scale from 0 (poor) to 100 (excellent) | Validated questionnaire |
| EQ-5D-5L | Continuous | A score from the EQ-5D-5L assessing overall health status. | Scale from 0 (worst health) to 1 (best health) | Validated questionnaire |
| TSK-11 | Continuous | A score from the TSK-11 questionnaire measuring fear of movement and re-injury. | Scale from 11 to 44 | Validated questionnaire |
| HADS Depression Score | Continuous | A score measuring the severity of depression symptoms. | Scale from 0 (no distress) to 21 (extreme distress) | Validated questionnaire |
| HADS Anxiety Score | Continuous | A score measuring the severity of anxiety symptoms. | Scale from 0 (no distress) to 21 (extreme distress) | Validated questionnaire |
| BPI Pain Intensity | Continuous | Self-reported pain intensity on a scale of 0-10. | Scale from 0 (no pain) to 10 (worst pain) | Validated questionnaire |
| IES-R Score | Continuous | A score measuring post-traumatic stress symptoms. | Scale from 0 (no distress) to 88 (high distress) | Validated questionnaire |
| PSEQ | Continuous | A score measuring self-efficacy in managing pain. | Scale from 0 (not confident) to 30 (very confident) | Validated questionnaire |
| CPGS | Continuous | A score measuring the complexity of the patient’s pain condition. | Based on clinical judgment; higher = more complexity | Clinical assessment |
| **Trauma Variables** | | | | |
| Trauma Description | Categorical | Description of the type of trauma sustained (e.g., accident, assault). | Coded categories (e.g., 1=Accident, 2=Assault, etc.) | Clinical records |
| Upper Limb Injury | Binary | Indicates presence of an upper limb injury (yes/no). | Yes (1), No (0) | Clinical assessment |
| Lower Limb Injury | Binary | Indicates presence of a lower limb injury (yes/no). | Yes (1), No (0) | Clinical assessment |
| Back/Neck Injury | Binary | Indicates presence of a back or neck injury (yes/no). | Yes (1), No (0) | Clinical assessment |
| Chest/Abdominal Injury | Binary | Indicates presence of a chest or abdominal injury (yes/no). | Yes (1), No (0) | Clinical assessment |
| Head/Face Injury | Binary | Indicates presence of a head or face injury (yes/no). | Yes (1), No (0) | Clinical assessment |
| Number of Fractures | Count | Total number of fractures sustained. | Integer value | Clinical records |
| Days in Hospital | Continuous | Total number of days spent in the hospital. | Integer value | Clinical records |
| Number of Surgeries | Count | Total number of surgical procedures undergone. | Integer value | Clinical records |
| Injury Severity | Continuous | A score representing the severity of the injury (e.g., ISS score). | Scale (based on clinical assessment) | Clinical assessment |
| Previous Surgery | Binary | Indicates whether the patient has had previous surgeries (yes/no). | Yes (1), No (0) | Clinical records |
| Days Since Admission | Continuous | Number of days since the patient was admitted to the hospital. | Integer value | Clinical records |
| Days Since Trauma | Continuous | Number of days since the trauma occurred. | Integer value | Clinical records |
| **Socio-Demographics** | | | | |
| Civilian/Military | Categorical | Indicates whether the individual is a civilian or military member. | 1=Civilian, 2=Military | Self-reported/clinical records |
| Alcohol Consumption | Categorical | Indicates the level of alcohol consumption (e.g., none, moderate, heavy). | Coded categories (0=No, 1=Yes) | Self-reported questionnaire |
| Smoking Status | Categorical | Indicates smoking status (non-smoker, former smoker, current smoker). | Coded categories (0=No, 1=Yes) | Self-reported questionnaire |
| Drug Use | Categorical | Indicates usage of illegal drugs (yes/no). | Yes (1), No (0) | Self-reported questionnaire |
| Medical History | Categorical | Indicates the presence of significant medical history (yes/no). | Yes (1), No (0) | Clinical records |
| Comorbidities | Count | The number of additional health conditions diagnosed. | Yes (1), No (0) | Clinical assessment |
| Age | Continuous | Patient's age at the time of assessment. | Integer value (in years) | Clinical records |
| Gender | Categorical | Gender of the patient. | 1=Male, 2=Female | Self-reported questionnaire |
| Education Status | Categorical | Highest level of education completed (e.g., none, high school, college). | Coded categories (e.g., 0=None, 1=HS, 2=College, etc.) | Self-reported questionnaire |
| Work Status | Categorical | Indicates employment status (employed, unemployed). | Coded categories (1=Employed, 2=Unemployed) | Self-reported questionnaire |
| Ethnic Group | Categorical | Ethnic background of the patient. | Coded categories (e.g., 1=White, 2=Black, etc.) | Self-reported questionnaire |
