# Supplementary material for: Predictors of Post-Traumatic Stress Symptoms after musculoskeletal trauma
Source: PLoS One. 2026 May 6;21(5):e0348595. doi: 10.1371/journal.pone.0348595 (PMC13148695; doi:10.1371/journal.pone.0348595)
Supplement: S5 File — (DOCX) [file pone.0348595.s005.docx]

**Supplementary file 4: Collinearity testing at 6 months follow-up: Variance Inflation Factor (VIF)**

| **Variables** | **VIF** | **1/VIF** |
| --- | --- | --- |
| **CPGS pain** | ***9849.11*** | ***0.000102*** |
| **BPI pain intensity subscale** | ***9840.66*** | ***0.000102*** |
| **HADS depression** | 5.47 | 0.18 |
| **SF-36 (mental)** | 4.34 | 0.23 |
| **Pain Self Efficacy questionnaire (PSEQ)** | 4.32 | 0.23 |
| **HADS anxiety** | 4.24 | 0.23 |
| **EQ-5D-5L** | 3.13 | 0.31 |
| **CPGS disability** | 2.97 | 0.33 |
| **TSK-11** | 1.62 | 0.61 |

**Abbreviation**: HADS; Hospital Anxiety and Depression Scale; SF; Short Form; BPI; Brief Pain Inventory; TSK; Tampa Scale of Kinesiophobia; PSEQ; Pain Self Efficacy Questionnaire; CPGS; Chronic Pain Grade Scale.
