# Supplementary material for: Predictors of Post-Traumatic Stress Symptoms after musculoskeletal trauma
Source: PLoS One. 2026 May 6;21(5):e0348595. doi: 10.1371/journal.pone.0348595 (PMC13148695; doi:10.1371/journal.pone.0348595)
Supplement: S6 File — (DOCX) [file pone.0348595.s006.docx]

**Supplementary file 5: PCA results (6 months)**

| Component | Eigenvalues | Difference | Proportion | Cumulative |
| --- | --- | --- | --- | --- |
| HADs Depression | 5.98 | 4.90 | 0.67 | 0.67 |
| HADs Anxiety | 1.08 | 0.39 | 0.12 | 0.78 |
| SF-36 mental | 0.68 | 0.27 | 0.07 | 0.86 |
| EQ-5D-5L | 0.41 | 0.09 | 0.04 | 0.90 |
| Pain intensity | 0.32 | 0.07 | 0.03 | 0.94 |
| TSK-11 | 0.25 | 0.10 | 0.02 | 0.97 |
| CPGS Disability | 0.14 | 0.01 | 0.01 | 0.98 |
| CPGS Pain | 0.12 | 0.12 | 0.01 | 1.00 |
| PSEQ | 0.0005 | 0.00 | 0.00 | 1.00 |

**Abbreviation:** HADS; Hospital Anxiety and Depression Scale; SF; Short Form; BPI; Brief Pain Inventory; TSK; Tampa Scale of Kinesiophobia; PSEQ; Pain Self Efficacy Questionnaire; CPGS; Chronic Pain Grade Scale.

**Interpretation:**

HADs depression has the highest eigenvalue (5.98), indicating it explains the most variance among the components. HADs anxiety also contributes significantly but to a lesser extent than depression, with an eigenvalue of 1.08. Other components like SF-36 mental, EQ-5D-5L, Pain intensity, TSK-11, CPGS Disability, and CPGS Pain contribute modestly to the variance, with eigenvalues ranging from 0.68 to 0.12. The last two components, PSEQ and CPGS Pain, have minimal eigenvalues, suggesting they contribute little to the overall variance. As for the cumulative proportions, the first two components, HADs Depression and HADs Anxiety, already explain 78% of the total variance. Adding more components gradually increases the cumulative variance explained, with all components combined explaining 100% of the variance. Overall, this analysis suggests that depression and anxiety (measured by HADs Depression and HADs Anxiety) are the most significant factors influencing the observed data, followed by other factors contributing incrementally to the variance.
