# Supplementary material for: Predictors of Post-Traumatic Stress Symptoms after musculoskeletal trauma
Source: PLoS One. 2026 May 6;21(5):e0348595. doi: 10.1371/journal.pone.0348595 (PMC13148695; doi:10.1371/journal.pone.0348595)
Supplement: S7 File — (DOCX) [file pone.0348595.s007.docx]

**Supplementary file 6-9**

- **3 months follow-up**

1. **Mild and moderate injury severity**

| **Hypothesis Test Summary** | | | | |
| --- | --- | --- | --- | --- |
|  | Null Hypothesis | Test | Sig.^a,b^ | Decision |
| 1 | The distribution of PTSS_scores is the same across categories of Level_of_injury_severity. | Independent-Samples Mann-Whitney U Test | .075^c^ | Retain the null hypothesis. |
| a. The significance level is .050. | | | | |
| b. Asymptotic significance is displayed. | | | | |
| c. Exact significance is displayed for this test.  The results of the Independent-Samples Mann-Whitney U Test indicate that there is no statistically significant difference in the distribution of PTSS scores between the mild and moderate injury severity groups (p = .075). Since the p-value exceeds the significance level of .050, the null hypothesis, which states that the distributions are the same across the categories, is retained. This suggests that the level of injury severity does not have a significant effect on PTSS scores in this sample, indicating similar psychological distress levels among participants with mild and moderate injuries. | | | | |

1. **Mild and major injury severity**

| **Hypothesis Test Summary** | | | | |
| --- | --- | --- | --- | --- |
|  | Null Hypothesis | Test | Sig.^a,b^ | Decision |
| 1 | The distribution of PTSS_scores is the same across categories of Level_of_injury_severity. | Independent-Samples Mann-Whitney U Test | .356^c^ | Retain the null hypothesis. |
| a. The significance level is .050. | | | | |
| b. Asymptotic significance is displayed. | | | | |
| c. Exact significance is displayed for this test. | | | | |

The results of the Independent-Samples Mann-Whitney U Test indicate that there is no statistically significant difference in the distribution of PTSS scores between individuals with mild and major injury severity (p = .356). Since this p-value exceeds the significance level of .050, we retain the null hypothesis, which asserts that the distributions of PTSS scores are the same across these injury severity categories. This finding implies that the severity of injury, whether mild or major, does not significantly influence the levels of post-traumatic stress symptoms among the participants, indicating comparable psychological distress in both groups.

1. **Moderate and major injury**

| **Hypothesis Test Summary** | | | | |
| --- | --- | --- | --- | --- |
|  | Null Hypothesis | Test | Sig.^a,b^ | Decision |
| 1 | The distribution of PTSS_scores is the same across categories of Level_of_injury_severity. | Independent-Samples Mann-Whitney U Test | .857^c^ | Retain the null hypothesis. |
| a. The significance level is .050. | | | | |
| b. Asymptotic significance is displayed. | | | | |
| c. Exact significance is displayed for this test. | | | | |

The results of the Independent-Samples Mann-Whitney U Test show that there is no statistically significant difference in the distribution of PTSS scores between individuals with moderate and major injury severity (p = .857). As this p-value is greater than the significance level of .050, we retain the null hypothesis, which states that the distributions of PTSS scores are the same across these injury severity categories. This outcome indicates that the severity of injury, whether moderate or major, does not significantly impact the levels of post-traumatic stress symptoms in the participants, suggesting similar psychological distress among both groups.

- **6 months follow-up**

1. **Mild and moderate injury severity**

| **Hypothesis Test Summary** | | | | |
| --- | --- | --- | --- | --- |
|  | Null Hypothesis | Test | Sig.^a,b^ | Decision |
| 1 | The distribution of 6_Months_PTSS is the same across categories of injury_severity_score_category. | Independent-Samples Mann-Whitney U Test | .018^c^ | Reject the null hypothesis. |
| a. The significance level is .050. | | | | |
| b. Asymptotic significance is displayed. | | | | |
| c. Exact significance is displayed for this test. | | | | |

The results of the Independent-Samples Mann-Whitney U Test indicate that there is a statistically significant difference in the distribution of 6 Months PTSS scores between individuals with mild and moderate injury severity (p = .018). Since this p-value is less than the significance level of .050, the null hypothesis is rejected, which indicates that the distributions of 6 Months PTSS scores are the same across these injury severity categories. This finding suggests that the level of injury severity, specifically between mild and moderate, has a significant impact on the levels of post-traumatic stress symptoms experienced by the participants.

1. **Mild and major injury severity**

| **Hypothesis Test Summary** | | | | |
| --- | --- | --- | --- | --- |
|  | Null Hypothesis | Test | Sig.^a,b^ | Decision |
| 1 | The distribution of 6_Months_PTSS is the same across categories of injury_severity_score_category. | Independent-Samples Mann-Whitney U Test | .027^c^ | Reject the null hypothesis. |
| a. The significance level is .050. | | | | |
| b. Asymptotic significance is displayed. | | | | |
| c. Exact significance is displayed for this test.  The results of the Independent-Samples Mann-Whitney U Test reveal that there is a statistically significant difference in the distribution of 6 Months PTSS scores between individuals with mild and major injury severity (p = .027). Given that this p-value is less than the significance level of .050, the null hypothesis is rejected, which indicates that the distributions of 6 Months PTSS scores are the same across these injury severity categories. This finding indicates that the level of injury severity, specifically when comparing mild and major, significantly influences the levels of post-traumatic stress symptoms experienced by the participants | | | | |

1. **Moderate to major injury severity**

| **Hypothesis Test Summary** | | | | |
| --- | --- | --- | --- | --- |
|  | Null Hypothesis | Test | Sig.^a,b^ | Decision |
| 1 | The distribution of 6_Months_PTSS is the same across categories of injury_severity_score_category. | Independent-Samples Mann-Whitney U Test | .538^c^ | Retain the null hypothesis. |
| a. The significance level is .050. | | | | |
| b. Asymptotic significance is displayed. | | | | |
| c. Exact significance is displayed for this test. | | | | |

The results from the Independent-Samples Mann-Whitney U Test show that there is no statistically significant difference in the distribution of 6 Months PTSS scores between individuals with moderate and major injury severity (p = .538). Given that this p-value is greater than the significance level of .050, we retain the null hypothesis, which posits that the distributions of 6 Months PTSS scores are the same across the categories of injury severity. This suggests that the severity of injury, specifically in the comparison between moderate and major injuries, does not appear to have a significant impact on the post-traumatic stress symptoms experienced by the participants.

***Pre and post injury analysis: 3-months follow.***

| **Hypothesis Test Summary** | | | | |
| --- | --- | --- | --- | --- |
|  | Null Hypothesis | Test | Sig.^a,b^ | Decision |
| 1 | The distribution of PTSS_scores is the same across categories of Level_injury_severity. | Independent-Samples Kruskal-Wallis Test | .172 | Retain the null hypothesis. |
| a. The significance level is .050. | | | | |
| b. Asymptotic significance is displayed. | | | | |

| **Independent-Samples Kruskal-Wallis Test Summary** | |
| --- | --- |
| Total N | 41 |
| Test Statistic | 3.524^a^ |
| Degree Of Freedom | 2 |
| Asymptotic Sig.(2-sided test) | .172 |
| a. The test statistic is adjusted for ties. | |

The independent-samples Kruskal-Wallis test indicates no significant difference in post-traumatic stress symptom (PTSS) scores across injury severity categories, leading to the retention of the null hypothesis.

***Pre and post injury analysis: 6-months follow.***

| **Hypothesis Test Summary** | | | | |
| --- | --- | --- | --- | --- |
|  | Null Hypothesis | Test | Sig.^a,b^ | Decision |
| 1 | The distribution of 6_Months_PTSS is the same across categories of injury_severity_score_category. | Independent-Samples Kruskal-Wallis Test | .022 | Reject the null hypothesis. |
| a. The significance level is .050. | | | | |
| b. Asymptotic significance is displayed. | | | | |

**6_Months_PTSS across injury_severity_score_category**

| **Independent-Samples Kruskal-Wallis Test Summary** | |
| --- | --- |
| Total N | 37 |
| Test Statistic | 7.595^a^ |
| Degree Of Freedom | 2 |
| Asymptotic Sig.(2-sided test) | .022 |
| a. The test statistic is adjusted for ties. | |

The independent-samples Kruskal-Wallis test reveals a significant difference in 6-month post-traumatic stress symptom (PTSS) scores across injury severity score categories, leading to the rejection of the null hypothesis.
