# Supplementary material for: Predictors of Post-Traumatic Stress Symptoms after musculoskeletal trauma
Source: PLoS One. 2026 May 6;21(5):e0348595. doi: 10.1371/journal.pone.0348595 (PMC13148695; doi:10.1371/journal.pone.0348595)
Supplement: Attachment — Submitted filename: Response to Reviewers.docx [file pone.0348595.s011.docx]

**Response to the Academic Editor and Reviewers**

**Dear Dr Sidiq and Reviewers,**

We would like to thank you for the time and expertise you dedicated to reviewing our manuscript entitled *“Predictors of Post-Traumatic Stress Symptoms After Musculoskeletal Trauma”* (Manuscript ID: PONE-D-25-65953). We appreciate the constructive and insightful comments provided, which have helped us improve the clarity, methodological transparency, and overall quality of the manuscript. We have carefully addressed all comments raised by the Academic Editor and the reviewers. A detailed, point-by-point response is provided below. All revisions have been incorporated into the revised manuscript, and changes are clearly highlighted in the tracked-changes version. We confirm that the manuscript has been revised to comply fully with PLOS ONE formatting and submission requirements.

We hope that the revisions satisfactorily address all concerns, and we look forward to your further consideration of our work.

Kind regards,
Ferozkhan Jadhakhan
(On behalf of all authors)

**Academic Editor Comments**

**Comment 1:**

Journal Requirements: When submitting your revision, we need you to address these additional requirements.

1. **Please ensure that your manuscript meets PLOS ONE's style requirements, including those for file naming. The PLOS ONE style templates can be found at**

<https://journals.plos.org/plosone/s/file?id=wjVg/PLOSOne_formatting_sample_main_body.pdf> and

<https://journals.plos.org/plosone/s/file?id=ba62/PLOSOne_formatting_sample_title_authors_affiliations.pdf>

**Response to comment 1:**

Thank you for this important reminder. We have carefully reviewed the manuscript to ensure full compliance with PLOS ONE formatting and style requirements. The manuscript has been reformatted according to the journal template, including correct author and affiliation listing, title page structure, and overall layout. In addition, the abstract has been revised to comply with PLOS ONE’s structured abstract requirements and word limit.

Specific formatting revisions include updates to:

- Author and affiliation presentation
- Structured abstract formatting **(Pages 2-3, Lines 34-67)**
- Layout alignment with the PLOS ONE main body template
- File naming to conform to journal submission guidelines

We confirm that both the revised manuscript and the tracked-changes version reflect these updates.

**Comment 2:**

2. **We note that your Data Availability Statement is currently as follows: All relevant data are within the manuscript and in Supporting Information files.**

Please confirm at this time whether or not your submission contains all raw data required to replicate the results of your study. Authors must share the “minimal data set” for their submission. PLOS defines the minimal data set to consist of the data required to replicate all study findings reported in the article, as well as related metadata and methods (https://journals.plos.org/plosone/s/data-availability#loc-minimal-data-set-definition).

For example, authors should submit the following data:

- The values behind the means, standard deviations and other measures reported;
- The values used to build graphs;
- The points extracted from images for analysis.

Authors do not need to submit their entire data set if only a portion of the data was used in the reported study.

If your submission does not contain these data, please either upload them as Supporting Information files or deposit them to a stable, public repository and provide us with the relevant URLs, DOIs, or accession numbers. For a list of recommended repositories, please see https://journals.plos.org/plosone/s/recommended-repositories.

**Response to Comment 2: Data Availability**

Thank you for highlighting this requirement. We confirm that the minimal dataset required to reproduce the findings has been prepared and will be uploaded as Supporting Information alongside the revised manuscript.

The dataset includes the cleaned analytical data used to generate:

- All descriptive statistics (Table 1),
- Regression analyses,
- Model performance metrics,
- And statistical tests reported in the manuscript.

We confirm that the dataset contains the necessary values behind the reported means, standard deviations and regression outputs to ensure reproducibility. We have added a Data Availability subsection to the Methods section to clearly indicate where the minimal dataset is provided. The statement specifies that the dataset is included as Supporting Information (S5 file) and contains the information required to reproduce the study findings. This statement can be found in the revised manuscript in the Methods section **(page 13, lines 311-315).**

**Comment 2.1:**

If there are ethical or legal restrictions on sharing a de-identified data set, please explain them in detail (e.g., data contain potentially sensitive information, data are owned by a third-party organization, etc.) and who has imposed them (e.g., an ethics committee). Please also provide contact information for a data access committee, ethics committee, or other institutional body to which data requests may be sent. If data are owned by a third party, please indicate how others may request data access.

**Response to Comment 2.1: Ethical or Legal Restrictions on Data Sharing**

Thank you for your comment regarding data availability and potential restrictions. This study received ethical approval from the relevant research ethics committee (Approval number: 17/WA/0421). All data used in this secondary analysis were fully anonymised prior to access and analysis. No directly identifiable personal information was included in the dataset. We confirm that there are no ethical or legal restrictions preventing the sharing of the de-identified dataset. The data were collected with participant consent for research purposes, and anonymisation procedures were applied before analysis to ensure confidentiality. Therefore, the minimal dataset required to reproduce the findings can be shared in accordance with journal requirements.

**Comment 3:**

3. If the reviewer comments include a recommendation to cite specific previously published works, please review and evaluate these publications to determine whether they are relevant and should be cited. There is no requirement to cite these works unless the editor has indicated otherwise.

**Response to Comment 3: Suggested Additional Citations**

We have reviewed the manuscript to ensure appropriate citation of relevant literature. All referenced works are directly relevant to the study background, methods, and discussion, and no additional citations were deemed necessary.

**Comment 4:**

4. Please review your reference list to ensure that it is complete and correct. If you have cited papers that have been retracted, please include the rationale for doing so in the manuscript text, or remove these references and replace them with relevant current references. Any changes to the reference list should be mentioned in the rebuttal letter that accompanies your revised manuscript. If you need to cite a retracted article, indicate the article’s retracted status in the References list and also include a citation and full reference for the retraction notice. **[Note: HTML markup is below. Please do not edit.]**

**Response to Comment 4: Reference List Review**

Thank you for your comment regarding the reference list. We have carefully reviewed all cited references to ensure accuracy, completeness, and compliance with journal requirements. No cited references have been retracted, and no changes were required to the reference list during revision. Therefore, the reference list remains unchanged, as all sources cited are relevant and appropriate to support the manuscript.

**Reviewer 1: Comment**

1. Overall, the manuscript is scientifically sound and designed appropriately. The design of study, measurement tools and analytical outline are supporting the aims. It clearly defines the outcomes. However, there is minor suggestion in conclusion especially for inverse associations (e.g., anxiety, disability), should be interpreted more cautiously and framed as associative rather than protective effects.

**Response: Interpretation of Inverse Associations**

Thank you for your thoughtful comment. We agree that the inverse associations observed for anxiety and disability should be interpreted cautiously and framed as statistical associations rather than protective effects. We have revised the manuscript to reflect this clarification. Specifically, we added statements in the Discussion emphasising that:

- The findings are associative and do not imply causation.
- The observed inverse relationships do not indicate protective effects.
- Causal inferences cannot be drawn given the observational study design.
- Alternative explanations such as residual confounding, measurement timing, statistical artefact, and reverse causality are possible.

These clarifications have been added in:

- **Page 22, Lines 410-414**
- **Page 25, Lines 472-477** (within the subsection “Anxiety, Fear of Movement, and Disability”)
- **Page 28-29 Lines 564-567**

We believe these revisions now better reflect the appropriate level of caution in interpreting these findings.

2. **Clarification of the use of PCA within the regression models and acknowledgment of potential overfitting due to sample size would further strengthen the analysis.**

**Response: Managing multicollinearity in Regression Analysis**

Thank you for this helpful suggestion. We have clarified that PCA was used to reduce redundancy among correlated psychological and pain-related variables and to improve model stability. The retained components represent latent constructs and were entered into the regression models to reduce multicollinearity. **See pages 12, Lines 273-277** for clarity. We have now expanded the Strengths and Limitations section to acknowledge the potential risk of overfitting due to the modest sample size relative to the number of predictors. We also emphasise that findings should be interpreted cautiously and validated in larger independent samples **(page 27-28, lines 537-541)**

3. **Specify which supplementary files contain the underlying datasets and whether analysis code is available would improve transparency and reproducibility.**

**Response: Supplementary Files and Analysis Code**

Thank you for this helpful suggestion. We have clarified in the manuscript that the minimal underlying dataset is provided in Supplementary File 4 (S4 file). We have also provided the statistical analysis code as Supplementary File 5 (S5 file).

4. **Minor language editing is required and reduction of repetitions in the Methods and Results sections would improve clarity and conciseness.**

**Response: Clarity and conciseness**

Thank you for this helpful suggestion. We have carefully revised the manuscript to improve clarity and conciseness, including minor language editing and reduction of repetition in the Methods and Results sections.

**Reviewer 2: Comment**

1. **Clinical implications could be expanded, particularly regarding screening and early intervention strategies.**

**Response: Clinical Implications**

Thank you for the suggestion. We have expanded the Clinical Implications section to emphasise structured psychological screening in the early post-injury period and the use of validated tools such as the IES-R and HADS. We also highlight early referral to trauma-informed interventions. This revision appears on **Page 27, Lines 518-524.**

2. **Further explanation of the variable selection strategy would strengthen methodological clarity.**

**Response: Variable Selection Strategy**

Thank you for this suggestion. We have expanded the Methods section to provide a clearer explanation of our variable selection strategy. We now explicitly describe the a priori selection of predictors based on prior literature, the use of univariate screening (p<0.05), retention of clinically relevant confounders regardless of significance, and cautious application of backward elimination to minimise overfitting. **See page 10-11, Lines 244-250.**

3. **Minor grammatical and typographical corrections are recommended.**

Thank you for this comment. We have carefully reviewed the manuscript and corrected minor grammatical errors and typographical issues to improve clarity and readability throughout the revised version.

4. **Clarification of the variable selection strategies and PCA interpretation will improve transparency.**

**Response: Variable selection and PCA interpretation.**

Thank you for this comment. We have clarified the variable selection strategy and the interpretation of the PCA in the Methods section. The relevant revisions can be found on Pages **10-11 (Lines 244-250) and Page 12 (Lines 273-277),** respectively. We believe these additions improve transparency and methodological clarity.

5. **The unexpected inverse association between anxiety, kinesiophobia and PTSS requires more cautious interpretation and consideration of alternative explanation. Overall, this study quite valuable and can be a good contribution to trauma rehabilitation research.**

**Response: Inverse Associations**

Thank you for this constructive comment. We agree that the inverse associations between anxiety, kinesiophobia, and PTSS require cautious interpretation. We have revised the Discussion to emphasise that these findings are associative, do not imply protective effects, and cannot support causal inference. We also discuss alternative explanations. The relevant revisions can be found on **Page 22 (Lines 410-414), Page 25 (Lines 472-477), and Page 28-29 (Lines 564-567).**

**Administrative Updates**

The corresponding author has recently moved institution. We kindly request that all future correspondence regarding this manuscript be directed to: [ferozkhan.jadhakhan@bcu.ac.uk](mailto:ferozkhan.jadhakhan@bcu.ac.uk)

In addition, regarding the Article Processing Charge (APC), we request that the invoice be addressed to Professor Deborah Falla at the University of Birmingham, as the University of Birmingham holds an institutional APC agreement with PLOS ONE. This arrangement is necessary due to the corresponding author’s recent institutional move.
