# Supplementary material for: Predictors of Post-Traumatic Stress Symptoms after musculoskeletal trauma
Source: PLoS One. 2026 May 6;21(5):e0348595. doi: 10.1371/journal.pone.0348595 (PMC13148695; doi:10.1371/journal.pone.0348595)
Supplement: Attachment — Submitted filename: Response_to_Reviewers_auresp_2.docx [file pone.0348595.s012.docx]

**Response to Reviewers**

**Dear Dr Sidiq and Reviewers,**

We would like to thank you for the time and expertise you dedicated to reviewing our manuscript entitled *“Predictors of Post-Traumatic Stress Symptoms After Musculoskeletal Trauma”* (Manuscript ID: PONE-D-25-65953R1). We appreciate the constructive and insightful comments provided, which have helped us improve the clarity, methodological transparency, and overall quality of the manuscript. We have carefully addressed all comments raised by the Academic Editor and the reviewers. A detailed, point-by-point response is provided below. All revisions have been incorporated into the revised manuscript, and changes are clearly highlighted in the tracked-changes version. We confirm that the manuscript has been revised to comply fully with PLOS ONE formatting and submission requirements.

We hope that the revisions satisfactorily address all concerns, and we look forward to your further consideration of our work.

Kind regards,
Ferozkhan Jadhakhan
(On behalf of all authors)

**Reviewer #1: 1. Comments to the Author**

The revised manuscript has improved substantially in clarity, transparency, and methodological reporting. The authors have addressed several previous concerns, particularly regarding missing data handling, multicollinearity, and cautious interpretation of findings. The topic is clinically relevant, and the longitudinal design is appropriate. However, several important issues remain that need to be addressed before the manuscript can be considered fully suitable for publication.

**Major comments:**

1. **Risk of overfitting and model stability**

The sample size is relatively small, particularly at follow-up (n≈73 at 3 months; n≈82 at 6 months), in relation to the large number of candidate predictors included in the regression models. This raises concerns about overfitting and model instability. The authors should:

- Report the number of outcome events used in each model
- Provide an events-per-variable (EPV) estimate
- Temper claims regarding model performance (e.g., AUC = 0.91), as these may be optimistic without external validation.

**RESPONSE TO REVIEWER-COMMENT 1**

**Risk of overfitting and model stability**

We thank the reviewer for this important comment regarding potential overfitting, model instability, and the need for greater transparency in reporting model performance. We agree that the relatively small number of outcome events at follow-up, compared with the number of candidate predictors, may increase the risk of overfitting. We have therefore revised the manuscript to explicitly report the number of outcome events, calculate and present events-per-variable (EPV) for both models, and appropriately temper interpretation of model performance, including the AUC. These revisions have been incorporated across the Methods, Results, Discussion, and Limitations sections.

1. **Reporting of outcome events and EPV**

In response to the reviewer’s request, we now explicitly report the number of outcome events and EPV for both time points.

**Methods section (Page 12, Lines 292-297)**

We added:

- 3-month model: 33 PTSS events/10 predictors: EPV = 3.3
- 6-month model: 22 PTSS events/12 predictors: EPV = 1.8

Explicit statement that the 6-month model demonstrates particularly low EPV and potential overfitting risk

2. **Acknowledgement of exploratory modelling approach**

**Methods section (Page 12, Lines 274-279)**

We clarified the modelling framework as exploratory:

This approach was used in an exploratory modelling framework given the relatively low events-per-variable ratio, and results should therefore be interpreted as hypothesis-generating rather than confirmatory. This directly addresses concerns about model instability and over-interpretation.

3. **Reinforcement of EPV reporting in Results**

Results section **(Page 22-23, Lines 427-433)**

We expanded the model performance reporting to include:

- Number of events per model
- Predictors included in each model
- EPV calculations (3.3 and 1.8)
- Explicit caution regarding overfitting and instability

We also linked low EPV to potential optimism in model performance estimates.

4. **Tempering interpretation of model performance (AUC)**

Results section (Model Performance subsection)

**We revised the interpretation of the AUC:**

The model demonstrated high apparent discriminatory ability with an AUC of 0.91; however, this should be interpreted cautiously given the small number of outcome events, low events-per-variable ratio, and absence of external validation, all of which may lead to optimistic performance estimates. This directly addresses the reviewer’s concern regarding potentially inflated performance metrics.

**Strengthening limitations regarding EPV and overfitting**

Discussion/Limitations section **(Page 32, Lines 655-670)**

We further reinforced this issue in the limitations section:

A key limitation of this study is the low events-per-variable (EPV) ratio in the multivariable models, particularly at six months (EPV = 1.8), which falls below commonly recommended thresholds for stable logistic regression modelling. This increases the risk of overfitting and reduces confidence in the stability of regression coefficients and predictive performance metrics. Consequently, findings from the predictive models, including the AUC estimates, should be interpreted as exploratory and require validation in larger independent cohorts.

2. **Reviewer Comment 2: Interpretation of inverse associations (anxiety, kinesiophobia, disability). The finding that higher anxiety, kinesiophobia, and disability are associated with reduced odds of PTSS contradicts the broader literature. While the authors acknowledge this and provide possible explanations, these remain speculative. The interpretation should be further softened and clearly framed as hypothesis-generating rather than explanatory. Alternative explanations such as residual confounding, measurement timing, or statistical artifacts should be emphasized more strongly.**

**RESPONSE TO REVIEWER-COMMENT**

We thank the reviewer for this important and insightful comment. We agree that the observed inverse associations between anxiety, kinesiophobia, disability, and PTSS require cautious interpretation and should not be viewed as contradicting the wider literature or implying protective effects. In response, we have substantially revised the Discussion and Conclusion sections to further soften the interpretation, explicitly frame these findings as hypothesis-generating, and strengthen the emphasis on alternative explanations such as residual confounding, measurement timing effects, reverse causality, and statistical artefact due to small sample size and low events-per-variable (EPV).

**Manuscript changes made:**

1. **Discussion - Anxiety, Fear of Movement, and Disability section (Page 26-27, lines 515-546):**
   We have substantially revised the interpretation of these findings to ensure they are not presented as protective or causal effects.

These revisions now explicitly state that:

- The findings should be interpreted cautiously as hypothesis-generating associations rather than evidence of a protective effect.
- They may reflect study-specific or sample-specific associations rather than true inverse relationships.
- They should not be interpreted as contradicting established literature.

For example, the revised text at **Page 26, lines 515-516** now explicitly states:

that these findings “should be interpreted cautiously as hypothesis-generating associations rather than evidence of a protective effect.”

We further strengthened interpretation across **Pages 26-27 (lines 522-524)** by explicitly stating that:

- These associations may reflect residual confounding, measurement timing effects, or statistical artefact
- The findings are exploratory signals rather than confirmatory evidence
- Any mechanistic explanations (e.g., coping strategies, resilience) are speculative and cannot be confirmed within the observational design

2. **Limitation/Interpretation reinforcement (Page 32-33, lines 626-701):**
   We further strengthened the limitation and conclusion sections to reinforce caution in interpretation.

At **Page 32, lines 655-670**, we now explicitly state that:

- The inverse associations should be interpreted strictly as exploratory findings
- They are highly susceptible to residual confounding, measurement timing effects, reverse causality, and statistical artefact
- They should not be interpreted as protective effects or used to guide clinical decision-making
- Replication in larger, independent cohorts with adequate power and external validation is required

3. **Use of linear regression for a binary outcome. The manuscript states that linear regression was used with a binary PTSS outcome during initial analysis. This is not appropriate and should be corrected or clarified. If used only as a screening step, this should be explicitly justified.**

Thank you for highlighting this important methodological point. We agree that linear regression is not appropriate for modelling a binary outcome. In this study, linear regression was used solely as a preliminary exploratory screening step to examine crude associations between candidate predictors and PTSS, and not for inference or model estimation. All final analyses and reported results are based on binary logistic regression models, which are appropriate for a dichotomous outcome. This clarification has now been explicitly added to the Methods section (pages 10-11, lines 244-250 and page 11, lines 269-272), where we also emphasise that results from the linear regression step were not interpreted or presented.

4. **Insufficient detail on cross-validation. The manuscript mentions cross-validation but does not describe the method (e.g., k-fold, split-sample, bootstrap). This needs clarification, or the statement should be removed.**

Thank you for this comment. We agree that the original statement regarding cross-validation lacked sufficient detail and may have caused ambiguity. To ensure clarity and transparency, we have removed the reference to cross-validation from the manuscript. The Methods section has been revised to specify that model performance was evaluated using discrimination and calibration metrics, which are explicitly reported in the Results (AUC and Hosmer–Lemeshow test). This change has been made on **page 12, lines 283-285**.

5. **Baseline PTSS prevalence (97.6%). The extremely high baseline prevalence suggests a potential ceiling effect related to the chosen cut-off (IES-R ≥22) and timing of assessment. The authors should explicitly acknowledge how this may affect discrimination and interpretation of predictors.**

**Response:** We thank the reviewer for this important observation. We have now explicitly acknowledged the potential ceiling effect associated with the high baseline PTSS prevalence, which is likely influenced by the early timing of assessment and the use of a sensitive IES-R cut-off (≥22). We have clarified that this may reflect transient post-traumatic distress and may limit variability in the outcome, thereby potentially affecting model discrimination and the interpretation of predictor effects. These points have been incorporated into the Methods (page 7, lines 154-158), Abstract (page 2-3, lines 56-57), and Discussion (page 30, lines 605-612) sections.

**Minor comments:**

1. **Clarify the type of informed consent obtained (e.g., written).**

**Response:** Thank you for this comment. We have now clarified the type of informed consent obtained. Specifically, we have added that written informed consent was obtained from all participants prior to study inclusion. This has been included in the Methods section (page 5, lines 115-116).

2. **Improve formatting and clarity of tables (e.g., inconsistencies in Table 2)**

**Response:** Thank you for this helpful suggestion. We have revised Table 2 to improve clarity, consistency, and readability. Specifically, we corrected formatting inconsistencies in confidence intervals and p-values, standardised decimal places, removed an incorrectly reported coefficient, and ensured appropriate alignment of variables and reference categories. Similar improvements have been applied to other tables where necessary.

**Reviewer #2: Comments to the authors**

This is a well-written and clinically relevant study examining predictors of post-traumatic stress symptoms following musculoskeletal trauma. The longitudinal design and use of validated outcome measures are strengths, and the manuscript demonstrates improved transparency compared to earlier versions. However, there are several methodological and interpretative issues that should be addressed to strengthen the manuscript.

**Major comments:**

1. **Predictive modelling limitations**

The ratio of predictors to sample size appears high, particularly at follow-up timepoints, increasing the risk of overfitting. While the authors acknowledge this limitation, the strength of the reported model performance (e.g., high AUC) may be overstated. This should be more clearly contextualized, and the limitations expanded.

**Response:** We thank the reviewer for this important and insightful comment. We agree that the relatively high number of candidate predictors in relation to the number of outcome events, particularly at follow-up timepoints, increases the risk of model overfitting and may result in optimistic estimates of model performance. To address this, we have strengthened the manuscript to more clearly acknowledge and contextualise these limitations across multiple sections:

**In the Methods** **(page 12, lines 274-279),** we have explicitly stated the increased risk of overfitting due to the predictor-to-event ratio and noted the potential for model instability. In the Model Performance section **(page 14, lines 342-345),** we clarified that performance metrics, including AUC, should be interpreted cautiously given the low events-per-variable ratio and lack of external validation.

**In the Results** **(page 22-23, lines 427-433),** we revised the description of model performance to emphasise that the high AUC may be optimistic and influenced by overfitting.

**In the Discussion** **(page 25, lines 478-481),** we added contextual interpretation highlighting that the observed associations may be inflated due to model complexity relative to sample size.

**In the Strengths and Limitations section** **(page 32, lines 659-663),** we expanded the discussion to explicitly state that discrimination metrics such as AUC may be overestimated and may not generalise to external populations.

**Finally, in the Conclusion** **(page 33, lines 688-694),** we emphasised that the findings should be considered preliminary and require validation in larger, independent cohorts before clinical application.

These revisions ensure that the limitations related to overfitting, model stability, and potential overestimation of predictive performance are clearly and consistently communicated throughout the manuscript. We believe these changes have strengthened the transparency and interpretation of our findings in line with the reviewer’s recommendation.

2. **Use of PCA in regression modelling.**

While PCA is used to address multicollinearity, it reduces interpretability of the predictors. The authors should explicitly acknowledge this limitation and clarify how PCA-derived components relate to clinically meaningful constructs.

**Response:** We thank the reviewer for this valuable comment and agree that the use of Principal Component Analysis (PCA), while addressing multicollinearity, introduces challenges in terms of clinical interpretability. We have revised the manuscript to explicitly acknowledge and clarify this limitation:

**In the Methods section** **(page 13, lines 313-318),** we have clarified that PCA-derived components represent linear combinations of correlated variables rather than directly observable clinical measures. We have also added text to explain that these components were interpreted based on their dominant contributing variables (e.g., psychological distress and pain-related factors), allowing approximate mapping to clinically meaningful domains, while acknowledging that precise attribution to individual predictors is not possible.

**In the Discussion** (**pages 31, lines 634-641),** we have expanded the limitations to explicitly state that the use of PCA reduces the clinical interpretability of the findings, as regression coefficients relate to composite components rather than individual variables. We further emphasised that this limits direct translation into specific clinical targets and highlighted the trade-off between statistical stability and interpretability.

These revisions ensure that the limitations associated with PCA, particularly regarding interpretability and clinical applicability, are clearly acknowledged and appropriately contextualised.

3. **Unexpected direction of key associations**

The inverse relationship between anxiety, kinesiophobia, disability, and PTSS is counterintuitive. Although alternative explanations are discussed, these remain speculative and should be framed more cautiously. The possibility of bias, measurement issues, or model artifacts should be emphasized.

**Response:** We thank the reviewer for this important and insightful comment. We agree that the observed inverse associations between anxiety, kinesiophobia, disability, and PTSS are counterintuitive and require particularly cautious interpretation. In response, we have revised the manuscript to strengthen the framing of these findings and to more clearly emphasise potential sources of bias and methodological limitations:

**In the Results section (page 22-23, lines 427-433),** we have explicitly acknowledged that these inverse associations are unexpected and may reflect residual confounding, measurement timing effects, or statistical artefact rather than true underlying relationships.

**In the Discussion** **(page 24, lines 469-472**), we have added text early in the interpretation to highlight the counterintuitive nature of these findings and emphasise that they may be influenced by bias, measurement limitations, or model-related artefacts rather than representing protective effects. **We have further revised the Discussion** (page 25, lines 478-481) to reduce speculative interpretation and clarify that any proposed explanations remain uncertain and should not be interpreted as causal mechanisms. **Additionally, in the Discussion** **(page 32, lines 655-670)**, we have strengthened the limitations by explicitly noting that the combination of small sample size, low events-per-variable ratio, and correlated predictors increases the likelihood of model instability and artefactual associations.

These revisions ensure that the unexpected direction of the associations is clearly acknowledged and that their interpretation is appropriately cautious, with greater emphasis on potential bias, measurement limitations, and statistical artefact.

4. **Clarity of statistical methods**

**Greater clarity is needed regarding:**

- The role of univariate screening.
- The backward elimination process.
- The cross-validation approach

**Response to Reviewer:** We thank the reviewer for this valuable comment and agree that greater clarity regarding the statistical modelling strategy is important for transparency and reproducibility. We have revised the manuscript accordingly and provided additional detail in the Methods, Results, Discussion, and Conclusion sections.

**Specifically:**

**Methods (page 11-12, lines 243-290):** We clarified the role of univariate analyses as an initial screening step for candidate predictors, including the use of a significance threshold (p<0.05), while emphasising that clinically relevant variables were retained irrespective of statistical significance. We also expanded the description of the backward elimination process, specifying that variables were sequentially removed based on statistical criteria while monitoring model fit and retaining clinically meaningful predictors. In addition, we clarified the use of cross-validation techniques for internal validation and assessment of model stability.

**Results (page 22-23, lines 427-433):** We added clarification linking model performance results to the underlying modelling strategy, including the use of screening, variable selection, and internal validation procedures.

**Discussion (page 32, lines 655-670):** We expanded the limitations to explicitly acknowledge the potential biases associated with data-driven approaches such as univariate screening and backward elimination, including risks of model instability and overfitting, particularly given the small sample size and low events-per-variable ratio.

**Conclusion (page 33, lines 688-694):** We added a statement highlighting that the findings should be interpreted in light of the modelling strategy and emphasised the need for validation in larger, independent cohorts using prespecified models. These revisions improve the transparency of the statistical approach and ensure that the modelling process is clearly described and appropriately contextualised.

5. **Generalizability**

The exclusion criteria (e.g., neurological conditions, severe cognitive impairment) and single-center design may limit generalizability. This should be discussed more explicitly

**Response to Reviewer:** We thank the reviewer for this important comment. We agree that the exclusion criteria and single-centre design may limit the generalisability of the findings, and we have revised the manuscript to address this more explicitly.

**Methods (page 6, lines 137-139):** We added a statement acknowledging that the exclusion criteria were applied to ensure a more homogeneous sample but may limit the applicability of the findings to broader trauma populations, particularly individuals with neurological conditions or cognitive impairment.

**Discussion (page 32, lines 647-652):** We expanded the limitations section to explicitly state that excluding patients with neurological conditions and severe cognitive impairment may reduce generalisability, as these groups may also be at increased risk of PTSS. We further emphasised that the single-centre design may limit external validity across different healthcare systems and trauma populations.

**Conclusion (page 34, lines 700-701):** We added a statement highlighting that caution is required when generalising findings beyond similar clinical settings and that further multi-centre studies are needed to confirm external validity.

**Minor Comments:**

- Clarify the consent procedure in the ethics statement.

**Response:** The consent procedure has been updated **page 5 line 115-116**

- Consider simplifying large tables for readability.

**Response:** Thank you for pointing this out, tables have been checked for inconsistencies and updated accordingly.
